# Chemerin 15 peptide reduces neuroinflammation via the ChemR23 receptor after ischemia–reperfusion injury

**DOI:** 10.4103/NRR.NRR-D-24-00137

**Published:** 2024-09-06

**Authors:** Yan Huang, Shuang Li, Yuhan Yang, Kunyi Li, Lan Wen, Jinglun Li

**Affiliations:** 1 Department of Neurology, The Affiliated Hospital of Southwest Medical University, Luzhou, Sichuan Province, China; 2 Laboratory of Neurological Diseases and Brain Function, The Affiliated Hospital of Southwest Medical University, Luzhou, Sichuan Province, China; 3 Department of Neurology, Clinical Medical College and the First Affiliated Hospital of Chengdu Medical College, Chengdu, Sichuan Province, China

**Keywords:** 5ʹ-monophosphate-activated protein kinase (AMPK), chemerin 15, chemerin, ChemR23, ischemia–reperfusion injury, microglia, neuroinflammation, neuronal apoptosis, stroke, nuclear factor kappa B (NF-κB)

## Abstract

Microglia-mediated neuroinflammation plays a crucial role in ischemic stroke; consequently, understanding its regulation could facilitate the development of therapies for ischemic stroke. Chemerin 15, a 15-amino acid peptide derived from chemerin, exerts powerful anti-inflammatory effects through ChemR23, modulates macrophage polarization, and diminishes inflammatory cytokine expression in peripheral inflammation models. However, its effects on microglia and stroke remain unclear. In this study, we used an *in vitro* oxygen/glucose deprivation BV2 cell model and a mouse model of ischemia-reperfusion injury to investigate the role of chemerin 15 in stroke and the underlying mechanisms. We co-cultured BV2 microglial cells with HT-22 hippocampal neurons and observed that chemerin 15 reduced apoptosis in HT-22 cells. Furthermore, we found that chemerin 15 binds to the ChemR23 receptor on the cell surface, inducing its internalization. This process regulated the activity of adenosine 5ʹ-monophosphate-activated protein kinase and inhibited its downstream target nuclear factor kappa B. These effects could be reversed by treatment with α-NETA, a ChemR23 inhibitor. In mice with ischemia-reperfusion injury, chemerin 15 modulated microglial polarization, reduced infarct volume and neuronal apoptosis, and facilitated cognitive and neurological function recovery. Our findings suggest that chemerin 15 suppresses the microglia-mediated inflammatory response, decreases neuronal apoptosis, and enhances long-term neurological function recovery by inducing ChemR23 internalization and regulating the adenosine 5ʹ-monophosphate-activated protein kinase/nuclear factor kappa B signaling pathway.

## Graphical abstract

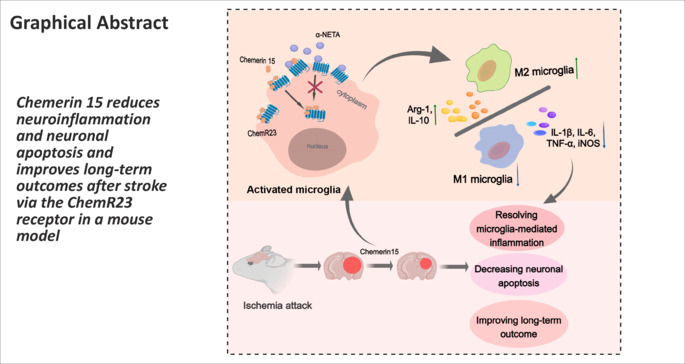

## Introduction

Intravenous thrombolysis and mechanical thrombectomy are recanalization therapies used to treat acute ischemic stroke (Mendelson and Prabhakaran, 2021). However, successful recanalization does not indicate a favorable clinical outcome, which may be related to the no-reflow phenomenon, microcirculatory disturbance, reperfusion injury, or cerebral edema (Sperring et al., 2023). Therefore, understanding these pathophysiological mechanisms will contribute to the development of effective treatment strategies.

The post–ischemic inflammatory response is initiated and amplified by damaged tissue and dead neural cells and characterized by microglial activation, subsequent recruitment of peripheral immune cells, and release of cytokines and chemokines (Chamorro et al., 2012; DeLong et al., 2022). Imaging and postmortem studies suggest that inflammation is not restricted to the site of primary ischemia, but rather spreads throughout the whole brain and persists for a prolonged time (Shi et al., 2019). During the acute and chronic phases of ischemic stroke, microglia are first responders that are swiftly activated and converge at the injury site, where their protein expression profiles change, shifting to a pro-inflammatory (M1) and/or protective (M2) phenotype (Cao et al., 2023). Consistent with their adopted phenotype, microglia play both positive (e.g., tissue remodeling and resolution of inflammation) and negative (e.g., tissue injury and pro-longed inflammation) roles after ischemic injury. Therefore, a balance between the two phenotypes must be maintained to ensure the effective removal of inflammatory insults while avoiding overly damaging or persistent inflammatory responses.

Higher organisms have evolved an endogenous anti-inflammatory network and a pro-resolving pathway that control the magnitude, duration, and location of the inflammatory response and promote tissue homeostasis. Resolution of inflammation is driven by special endogenous chemical mediators, such as proteins (e.g., chemerin and its derived peptides) and lipids (e.g., lipoxins, resolvins, and protectins) (Serhan and Levy, 2018; Feehan and Gilroy, 2019).

Chemerin is a 163–amino acid precursor protein with low biological activity that requires further processing at the C-terminus to form active fragments (Kennedy and Davenport, 2018). Under physiologic conditions, chemerin regulates adipocyte development and metabolic function as well as glucose metabolism. Recently, experimental evidence supports a role for chemerin in inflammation, obesity, and insulin resistance (Lin et al., 2014; Mariani and Roncucci, 2015; Su et al., 2021). Chemerin and its different molecular fragments exhibit varying activities and functions. Chemerin 9 (C9) and chemerin exerts an anti-inflammatory and pro-inflammatory effect. Chemerin 13 (C13) and chemerin 19 (C19) moderately suppress the inflammatory response, whereas chemerin 11 (C11) is devoid of anti-inflammatory activity (Cash et al., 2008). C11 and C13 possessed little macrophage chemotactic activity (Cash et al., 2008). The chemerin 15 fragment (C15; A140–A154; AGEDPHGYFLPGQFA), a 15-amino acid peptide derived from chemerin, has potent anti-inflammatory effects and pro-resolving properties (Cash et al., 2008). C15 is active at very low concentrations (in the picomolar range) and can polarize macrophages to adopt the M2 phenotype, inhibit the expression of pro-inflammatory mediators, and enhance macrophage-mediated phagocytosis in a ChemR23-dependent manner (Cash et al., 2010, 2014; Chang et al., 2015). ChemR23, also called chemokine-like receptor 1, is a chemoattractant receptor expressed by fat cells, dendritic cells, and peripheral and tissue-resident macrophages in mice and humans (Kaur et al., 2018; Kennedy and Davenport, 2018). It is reported that subcellular location of ChemR23 is on the surface of immune cells in human. ChemR23 is a class A G-protein coupled receptor, regulating adenylyl cyclase and subsequent cyclic adenosine monophosphate accumulation, intracellular calcium release, AMPK, and phosphorylation of mitogen-activated protein kinases (Kennedy and Davenport, 2018).

However, the effect of C15 on microglia and underlying mechanism during neuroinflammation following ischemic stroke remains unknown. We hypothesized that C15 regulates microglia-induced neuroinflammation and neuronal protection in cerebral ischemia–reperfusion (I/R) injury. To test this hypothesis, we used an *in vitro* oxygen-glucose deprivation (OGD) BV2 cell model and a well-established mouse model of I/R injury to determine the role of C15 and explore the underlying mechanisms.

## Methods

### C15 peptide synthesis

C15 was synthesized by Sicierbio Co. LTD. (Anhui, China), dissolved in 1 mM in phosphate buffered saline (PBS) and 0.1% bovine serum albumin (BSA), and stored at –80°C for up to 6 months. The peptide was freshly diluted for use in each experiment.

### Cell culture, oxygen-glucose deprivation model, and drug administration

BV2 murine microglial cells (ProCell, Wuhan, Hubei, China, Cat# CL-0493A) and HT-22 murine hippocampal neurons (ProCell, Cat# CL-0595) were cultured in Dulbecco’s modified Eagle’s high-glucose medium (Gibco, Grand Island, CA, USA) supplemented with 10% fetal bovine serum (Gibco) and 1% penicillin/streptomycin (Gibco). The cells were maintained at 37°C in a 5% carbon dioxide atmosphere, with the medium replaced every 2 days. When the cells reached 80%–90% convergence, they were digested with 0.25% trypsin (HyClone, Logan, UT, USA) and passaged for further subculture. Cells at passages 3–5 were used for biological tests.

OGD is an *in vitro* model that mimics I/R injury (Babu et al., 2022). Briefly, BV2 and HT-22 cells were transferred to glucose-free Dulbecco’s modified Eagle’s medium (ServiceBio, Wuhan, Hubei, China) and incubated at 37°C in a hypoxic incubator (94% N_2_, 1% O_2_, and 5% carbon dioxide). Subsequently, the cultured cells were returned to normal growth medium and cultured under the same conditions (including a normoxic gas mix) as described above. A Cell Counting Kit-8 (CCK-8) assay was conducted to assess cell viability and determine the timing of OGD. The cells in the control group were grown in high-glucose medium at 37°C in a 5% carbon dioxide atmosphere.

For the drug intervention group, BV2 cells were pretreated with α-NETA (2-[alpha-naphthoyl] ethyltrimethylammonium iodide, a ChemR23 inhibitor, 100 nM, MCE, Monmouth Junction, NJ, USA) for 24 hours and C15 (100, 250, 500, or 1000 ng/mL) for 30 minutes prior to OGD. Cells and supernatants from the different groups were collected for further experiments.

### Cell Counting Kit-8 assay

Cell viability was assessed using a CCK-8 kit (BOSTER, Wuhan, Hubei, China). All related experiments were performed according to the manufacturer’s instructions. Briefly, BV2 and HT-22 cells were plated at a concentration of 1.0 × 10^5^ cells/mL in 96-well culture plates and grown for 24 hours. After varying durations of OGD (1, 2, 3, and 4 hours), CCK-8 working solution (100 µL) was added to each well, and the plates were incubated for 1 hour at 37°C. The absorbance in each well was measured at 450 nm using a microplate reader (BioTek Cytation^TM^ 5; Agilent Technologies, Santa Clara, CA, USA).

### Real-time polymerase chain reaction

Total RNA from BV2 cells was extracted using TRIzol reagent (Invitrogen, Carlsbad, CA, USA), and spectrophotometric analysis (optical density_260 nm/280 nm_ > 1.8) was used to ensure the purity and quantity of the extracted RNA. Equal amounts of RNA (2 μg) were reverse-transcribed to complementary DNA using ExonScript RT SuperMix with dsDNase (Exongen, Chengdu, Sichuan, China). Real-time polymerase chain reaction analysis was conducted on a CFX96 Touch Real-Time PCR Detection System (Bio-Rad, Hercules, CA, USA) using Fast SYBR Green qPCR Master Mix UDG (Exongen, Chengdu, Sichuan, China) and corresponding primers. Glyceraldehyde-3-phosphate dehydrogenase (GAPDH) was used as an internal control. The mRNA levels of the target genes were normalized to that of GAPDH using the 2^–ΔΔCt^ method (Zhao et al., 2021) and are presented as relative expression units. The primer sequences are listed in **Table 1**.

**Table 1:** Sequences of the primers used in the study

Gene	Primer sequence (5'–3')	Product length (bp)
*IL-1β*	F: CAC CTC ACA AGC AGA GCA CAA G	22
	R: TTA GAA ACA GTC CAG CCC ATA CTT TAG	27
*TNF-α*	F: AAG ACA CCA TGA GCA CAG AAA GC	23
	R: GCC ACA AGC AGG AAT GAG AAG AG	23
*Arg-1*	F: AAG ACA GCA GAG GAG GTG AAG AG	23
	R: GAG TTC CGA AGC AAG CCA AGG	21
*GAPDH*	F: AAG TTC AAC GGC ACT GTC AAG G	22
	R: AAC ATA TTC GGC ACC AGC ATC AC	23

Arg-1: Arginase 1; F: forward; GAPDH: glyceraldehyde-3-phosphate dehydrogenase; IL: interleukin; R: reverse; TNF-α: tumor necrosis factor-α.

### Co-culture system and experimental design

To elucidate the indirect effects of C15 on neuronal apoptosis, we used a Transwell system (Corning, Corning, NY, USA) to co-culture BV2 cells with HT-22 cells. The Transwell system consisted of upper and lower compartments separated by a membrane with a pore size of 0.4 μm. BV2 cells (1 × 10^4^/100 µL/compartment) were placed in the upper compartment, while HT-22 cells (1 × 10^5^/500 µL/compartment) were placed in the lower compartment. Before being subjected to OGD, BV2 cells were pretreated with α-NETA for 24 hours and C15 for 30 minutes. The BV2 and HT-22 cells were then subjected to OGD for 3 hours and co-cultured for 4–6 hours. Finally, the HT-22 cells were harvested, and apoptosis was analyzed by flow cytometry.

### Flow cytometry

To detect the microglial phenotype and quantitatively analyze cell surface ChemR23 expression, BV2 cells were seeded into six-well plates at a density of 1 × 10^6^ cells/well and grown for 24 hours. The cells were then subjected to OGD for 3 hours, and the supernatant was collected to evaluate cytokine levels by enzyme-linked immunosorbent assay (ELISA). The BV2 cells were washed twice with PBS for 3 minutes and blocked with 0.1% BSA. Then, the cells were stained with a phycoerythrin (PE)-conjugated monoclonal mouse CD86 antibody (BioLegend, San Diego, CA, USA, Cat# 105007, RRID: AB_313150), BV421-conjugated monoclonal mouse CD206 antibody (BioLegend, Cat# 141717, RRID: AB_2562232), and Alexa Fluor 488–conjugated monoclonal mouse ChemR23 antibody (R D System, Minneapolis, MN, USA, Cat# FAB7610G, RRID: AB_3096974) at 4°C in the dark for 30 minutes, washed twice with PBS, and re-suspended in 350 µL PBS. Homologous isotype antibodies (PE, BV421, and Alexa Fluor 488–conjugated monoclonal antibodies) with irrelevant specificities were used as negative controls. All samples were examined using a BD LSRFortessa™ flow cytometer (BD Biosciences, San Jose, CA, USA). Flow Jo software (V10.6.2, FlowJo, LLC, Ashland, OR, USA) was used to perform the statistical analysis.

An Annexin V-APC/PI Cell Apoptosis Assay Kit (Elabscience, Wuhan, Hebei, China) was used to detect neuronal apoptosis. HT-22 cells were collected from the lower chamber of the co-culture system, washed twice with PBS, stained according to the manufacturer’s specifications, and analyzed using a BD LSRFortessa^TM^ flow cytometer (BD Biosciences) and Flow Jo (V10.6.2, FlowJo LLC).

### Animals

The animal experiments were approved by the Animal Research Ethic Committee of Southwest Medical University (Sichuan, China; approval No. swmu20230042) on April 6, 2023. Previous evidence indicates the estrogen, especially 17β-estradiol, mitigates ischemic injury after stroke (Vahidinia et al., 2020; Zhong et al., 2023), and thus only male mice were used in this study. A total of 170 adult C57/BL6J mice (male, 8–10 weeks old, 22–25 g) were obtained from the Animal Research Centre of the Southwest Medical University (Sichuan, China; license No. SYXK (Chuan) 2023-0065) and housed under standard conditions (25 ± 2°C, 65% ± 5% relative humidity, 12/12-hour light/dark cycle) with free access to food and water. All experiments were conducted in a blinded fashion, except for animal grouping and drug treatment. Data analysis was conducted by two investigators who were blind to the experimental group assignments.

### Transient middle cerebral artery occlusion/reperfusion model and drug administration

The cerebral I/R injury model is a line occlusion technique that induces transient middle cerebral artery occlusion/reperfusion (tMCAO/R). The mice were fasted for 1 day before surgery. An intraperitoneal injection of pentobarbital (TCI, Tokyo, Japan) was used to anesthetize the mice. Thereafter, a midline neck incision was made to expose the right common carotid artery, external carotid artery, and internal carotid artery. Subsequently, a monofilament (0.20 mm, Sinon Technology, Beijing, China) was inserted into the common carotid artery and slowly advanced into the internal carotid artery until the marker reached the carotid bifurcation and resistance was felt, indicating that the tip had reached the middle cerebral artery. After 1.5 hours of occlusion, the monofilaments were removed to allow reperfusion. All mice were euthanized using pentobarbital anesthesia (intraperitoneal administration, 150 mg/kg) at 0, 6, and 12 hours and 1, 3, 7, and 28 days following reperfusion (Zhao et al., 2020).

For the *in vivo* experiments, all drugs were prepared in normal saline. Briefly, α-NETA (10 mg/kg) was intraperitoneally injected after blood flow was blocked for 1 hour (Zhang et al., 2019), Intranasal administration of C15 (0.3 ng/kg) was performed immediately after reperfusion as described previously (Cash et al., 2008, 2010; Chang et al., 2015): alternating nostrils every 2 minutes (1–2 µL/administration), repeated every 12 hours.

### Western blot analysis

The mice were anesthetized by intraperitoneal injection of pentobarbital (150 mg/kg) and perfused with 50 mL normal saline, and the brains were carefully removed. BV2 cells or mouse brain tissues were treated with radioimmunoprecipitation assay lysis buffer (BOSTER) supplemented with protease inhibitors (BOSTER) and phosphatase inhibitors (BOSTER) on ice for 30 minutes and ultrasonically homogenized. A cell membrane/cytoplasm/nuclear membrane protein extraction kit (Solarbio, Beijing, China) was used to extract nuclear proteins, and the protein concentrations were quantified using a BCA Assay Kit (Beyotime, Nantong, Jiangsu, China). Next, equal amounts of protein (25 µg) from each group of cells or mice were separated by 10% polyacrylamide gel electrophoresis (Yamei, Shanghai, China) and transferred to a polyvinylidene fluoride membrane (Millipore, Burlington, MA, USA). The membrane was placed in a 5% BSA blocking solution for 2 hours, incubated with primary antibodies overnight at 4°C, and the following day incubated with a secondary antibody for 1 hour at 37°C. After each step, the membrane was washed three times for 5 minutes with tris-buffered saline containing Tween-20. The primary antibodies used for western blotting were as follows: anti–adenosine 5′-monophosphate-activated protein kinase α (AMPKα) rabbit antibody (1:1000, Cell Signaling Technology, Cat# 5832, RRID: AB_10624867), anti–phospho-AMPKα (Thr172) rabbit antibody (1:1000, Cell Signaling Technology, Cat# 50081, RRID: AB_2799368), anti–nuclear factor kappa-B (NF-κB) p65 recombinant rabbit monoclonal antibody (1:2000, Huabio, Cat# HA721307, RRID: AB_3072424), anti–ChemR23 rabbit polyclonal antibody (1:2000, Huabio, Cat# ER63003, RRID: AB_3096969), and anti–β-actin rabbit monoclonal antibody (1:2000, BOSTER, Wuhan, Hubei, China, Cat# BM3873, RRID: AB_3096973). The secondary antibody used was horseradish peroxidase (HRP)-conjugated goat anti–rabbit IgG antibody (1:5000, BOSTER, Cat# BA1054, RRID: AB_2734136). Protein bands were visualized using a ChemiDocTM MP Imaging System (Bio-Rad). The gray scale values of the bands were measured using ImageJ software (Version 1.54i, National Institutes of Health, Bethesda, MD, USA) (Schneider et al., 2012). β-Actin was used as an internal reference.

### 2,3,5-Triphenyltetrazolium chloride staining

Mice were euthanized by intraperitoneal injection of pentobarbital (150 mg/kg) at 24 hours post-reperfusion. The brain tissue was immediately removed, frozen at –20°C for 5 minutes, and cut into 2-mm coronal sections. The slices were incubated in 2% 2,3,5-triphenyltetrazolium chloride (BioFroxx, Saiguo Biotech Co. LTD, Guangzhou, China) at 37°C in the dark for 15 minutes and fixed with 4% paraformaldehyde for 2 hours. The infarction volume was calculated using ImageJ software and the following formula: (infarcted volume/whole brain volume) × 100%.

### Immunofluorescence staining

Paraffinized brain sections (3 µm) were deparaffinized and rehydrated, and antigen retrieval was performed. Subsequently, brain sections and BV2 cells (cultured on coverslips) were washed three times with PBS, permeabilized with 0.1% Triton X-100 for 30 minutes, blocked with 1% BSA for 1 hour at 4°C, and incubated with primary antibodies at 4°C overnight. The primary antibodies used for immunofluorescence staining were as follows: anti-CD86 rat monoclonal antibody (1:200, Thermo Fisher Scientific, Waltham, MA, USA, Cat# 14-0862-82, RRID: AB_467368), anti-CD206 goat polyclonal antibody (1:200, Thermo Fisher Scientific, Cat# PA5-46994, RRID: AB_2607366), anti–ionized calcium binding adaptor molecule 1 (IBA1) rabbit polyclonal antibody (1:200, Cell Signaling Technology, Danvers, MA, USA, Cat# 17198, RRID: AB_2820254), anti-ChemR23 rabbit polyclonal antibody (1:200, Huabio, Hangzhou, Zhejiang, China, Cat# ER63003, RRID: AB_3096969), anti-NeuN recombinant rabbit monoclonal antibody (1:200, Huabio, Cat# ET1602-12, RRID: AB_3069624). After rinsing twice with PBS, the sections were stained with secondary antibodies in the dark for 1 hour at 37°C. The secondary antibodies used were as follows: fluorescein isothiocyanate (FITC)-conjugated rabbit anti-goat IgG antibody (1:200, Huabio, Cat# HA1007, RRID: AB_3096970), Alexa Fluor 647–conjugated goat anti-rabbit IgG antibody (1:200, Huabio, Cat# HA1106, RRID: AB_3096971), FITC-conjugated goat anti-rabbit IgG antibody (1:200, Huabio, Cat# HA1124, RRID: AB_3096972), and Alexa Fluor 488–conjugated goat anti-rat IgG antibody (1:200, Cell Signaling Technology, Cat# 4416, RRID: AB_10693769). Nuclei were counterstained with 4,6-diamidino-2-phenyl-indole (1:1000, Absin, Shanghai, China) for 15 minutes, and the slices were rinsed again and imaged using a confocal laser scanning microscope (Leica, Wetzlar, Germany).

To assess neuronal apoptosis, brain sections were stained with an anti-NeuN antibody to detect neuronal nuclei and subjected to terminal deoxynucleotidyl transferase–mediated dUTP nick-end labeling (TUNEL) using a TUNEL Assay kit (BOSTER) according to the manufacturer’s instructions. 4,6-Diamidino-2-phenyl-indole was used to stain the nuclei. The samples were imaged using confocal laser scanning microscope, and the number of NeuN^+^TUNEL^+^ cells in each field was determined using ImageJ software.

### Enzyme-linked immunosorbent assay

After the brain tissue was removed as described above, the area of cerebral infarction was excised and homogenized in PBS. The homogenate was then centrifuged at 1000–1500 × *g* for 10 minutes, and the supernatant was removed and centrifuged at 800 × *g* at 4°C for 10 minutes. The concentrations of various cytokines, including IL-1β, IL-6, inducible nitric oxide synthase (iNOS), TNF-α, IL-10, and Arg-1, were measured using ELISA kits (JINGMEI BIOTECHNOLOGY, Yancheng, Jiangsu, China) following the manufacturer’s instructions. A microplate absorbance reader (DeTie, Nanjing, China) was used to detect absorbance at 450 nm. Standard curves were used to calculate the concentrations of the cytokines.

### Neurological function assessment

Several behavioral tests were used to assess the neurological function of mice at 1, 3, 7, 14, 21, and 28 days following I/R injury. Mice from different groups were trained for 3 days prior to injury. The data from the last training session were recorded as the baseline values.

The corner turn test was used to examine sensorimotor and postural asymmetry (Wang et al., 2016). To do this, the mice were positioned face-forward in 30° corner formed by two plastic plates, which required the subject to turn left or right to exit the corner. Uninjured mice turned to either side with the same frequency. The trial was repeated 10 times for at least 30 seconds, during which each mouse was allowed to turn once. The corner turn test results (%) were calculated as follows: (number of right turns/total number of turns) × 100.

The foot fault test was conducted to evaluate forelimb motor function (Liu et al., 2016). A stainless steel mesh platform (20 cm × 40 cm, with a 4 cm^2^ grid size) was placed 1 m above the ground. The mouse was placed on the mesh platform, and the total number of times that a forelimb fell through the grid (defined as a foot fault) during 1 minute was recorded. The foot fault results (%) were calculated as follows: (number of foot faults/otal number) × 100.

The Modified Neurological Severity Score (mNSS) system was used to assess neurological deficits, including motor and sensory functions, reflexes, and balance. Uninjured mice have a score of 0 points, while 1–6 points indicates mild damage, 7–12 moderate damage, and 13–18 severe damage (Ding et al., 2022; Zhou et al., 2023).

The Morris water maze test was conducted to assess cognitive function (Jia et al., 2023; Li et al., 2024). The pool (120 cm in diameter and 45 cm in height) was filled with water at a temperature of 21–22°C, and a platform was placed 1 cm below the surface of the water. High-contrast spatial cues were placed surrounding the pool to help mice recall where the platform was located. All mice were pre-trained twice a day for 23–27 days after injury. Each training period lasted 60 seconds. If the mouse found the platform within 60 seconds, the time and swimming speed were recorded; otherwise, they were manually guided to the platform and allowed to stay on it for 15 seconds, and the time to find the platform was recorded as 60 seconds. Twenty-four hours after the last training session (on day 28), the platform was removed from the pool, and the probe test was conducted within 60 seconds. The mice were placed in the water on the opposite side of the platform, and their swimming trajectories were recorded using a video system (Ethovision XT, NOLDUS, Wageningen, Netherland). The escape latency (the time to find the platform during training period), the number of times the mouse crossed the target area during the probe test, time in the target area, and average swimming speed were recorded.

### Statistical analysis

We used a resource equation approach to calculate the sample size. Taking into consideration the possibility of animal mortality, we have determined that the starting sample size should be 170. The data were analyzed by two investigators who were blinded to the experimental group assignments. Statistical analysis was performed using GraphPad Prism version 10.0.0 for Windows (GraphPad Software, Boston, MA, USA; www.graphpad.com). Before statistical analysis was performed, a quantile-quantile plot was used to assess the normality of the data, while Brown-Forsythe test and Bartlett’s test were performed to test group variance. Two-tailed *t*-test was applied to compare two groups. Repeated measurement analysis of variance and one-way analysis of variance with *post hoc* Tukey’s multiple comparison test were performed to compare multiple groups. Data are expressed as mean ± standard deviation (SD) for each sample, and statistically significance was defined as *P* < 0.05.

## Results

### Microglial phenotype and function change dynamically after oxygen-glucose deprivation

**Figure 1:**
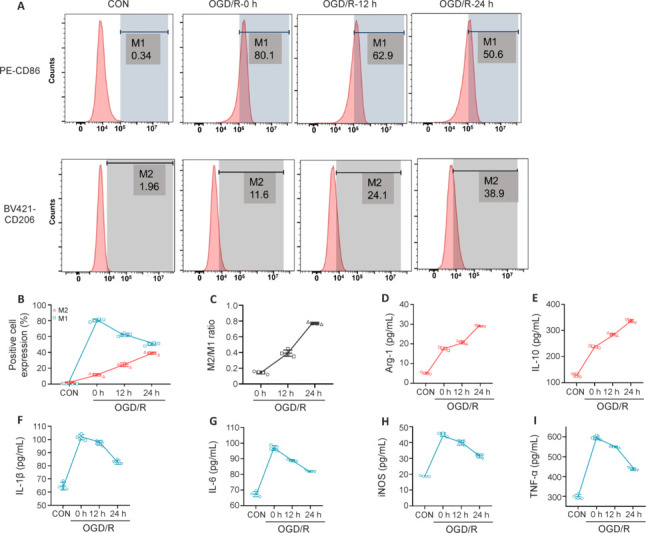
Changes in microglial phenotype and function following oxygen-glucose deprivation. (A) Representative flow cytometric histograms of BV2 cell polarization following reperfusion for 0, 12, and 24 hours. As the duration of reperfusion increased, the percentage of M1 microglia (CD86^+^ cells) decreased, while that of M2 microglia (CD206^+^ cells) increased. (B) Broken line graph of the percentages of CD86^+^ cells and CD206^+^ cells (*n* = 4 independent samples). (C) The ratio of M2 to M1 microglia. (D–I) Inflammatory cytokine expression as detected by ELISA. The expression levels of all cytokines increased after OGD/R, while the expression levels of pro-inflammatory factors (IL-1β, IL-6, iNOS, and TNF-α) decreased and anti-inflammatory factors (Arg-1 and IL-10) increased as reperfusion duration increased. Data are presented as the mean ± SD (*n* = 4 independent samples) and were analyzed using one-way analysis of variance with *post hoc* Tukey’s multiple comparison test. Arg-1: Arginase 1; CON: control; ELISA: enzyme-linked immunosorbent assay; IL: interleukin; iNOS: inducible nitric-oxide synthase; OGD/R: oxygen glucose deprivation-reperfusion; TNF-α: tumor necrosis factor-α.

First, we determined the optimal OGD duration via CCK-8 assay. The results showed a time-dependent decrease in cell viability in the OGD group. After 3 hours of OGD, the cell viability decreased to approximately 50%; therefore, we selected this duration for subsequent experiments (**Additional Figure 1**). Next, we used flow cytometry to identify the phenotype of the BV2 cells subjected to OGD. As shown in **Figure 1A–C**, nearly 80% of microglia were CD86^+^ after OGD, indicating activation to the M1 phenotype. As the duration of reperfusion time increased, the percentage of CD86^+^ cells decreased, while that of CD206^+^ cells (M2 phenotype) increased.

The M2/M1 ratio increased with prolonged reperfusion. Although the number of CD206^+^ cells increased, CD86+ cells remained dominant during reperfusion. To validate this, using ELISA, we measured the levels of inflammatory factors (IL-1β, IL-6, TNF-α, and iNOS) expressed by M1 macrophages, as well as the levels of anti-inflammatory mediators (Arg-1 and IL-10) secreted by M2 macrophages (**Figure 1D–I**). Quantitative analysis revealed that the expression levels of these mediators increased after OGD, while the expression levels of the inflammatory factors decreased and those of the anti-inflammatory mediators increased with reperfusion time. These results indicated that dynamic changes occur in microglial phenotypes after ischemia: the pro-inflammatory phenotype was predominant, and the anti-inflammatory phenotype gradually increased after reperfusion.

### C15 inhibits the oxygen-glucose deprivation–induced inflammatory response and neuronal apoptosis in microglia

**Figure 2:**
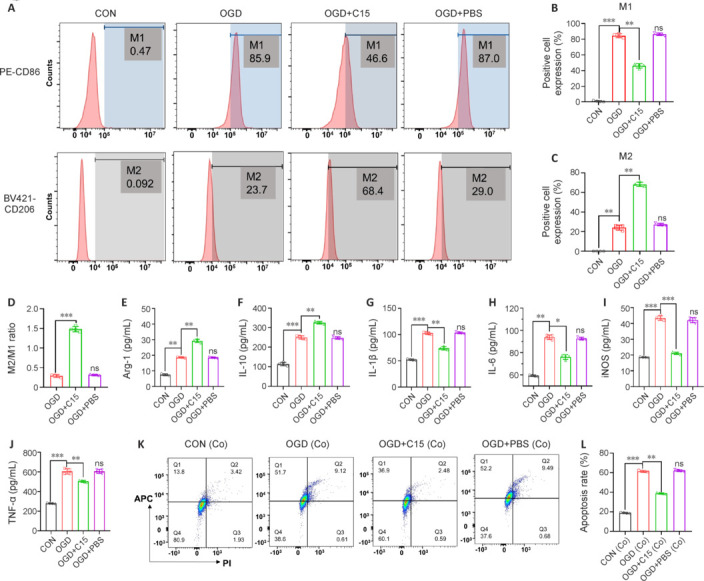
C15 regulates OGD-induced inflammation in microglia and decreases the apoptosis of neurons co-cultured with microglia. (A) Flow cytometric histograms of BV2 cell polarization. C15 treatment reduced the number of M1 microglia (CD86^+^ cells) and increased the number of M2 microglia (CD206^+^ cells). (B–D) Quantitative analysis of CD86^+^ (B) and CD206^+^ (C) cell numbers and the ratio of M2 to M1 microglia (D). (E–J) ELISA analysis demonstrated that C15 decreased expression of the pro-inflammatory factors IL-1β, IL-6, iNOS, and TNF-α and increased expression of the anti-inflammatory factors Arg-1 and IL-10. (K) Representative dot plots of apoptosis of HT-22 cells co-cultured with BV2 cells, as detected by flow cytometry. Treatment with C15 decreased the proportion of apoptotic neurons. (L) Statistical analysis of HT-22 cell apoptosis in the different groups. Data are presented as the mean ± SD (*n* = 4 independent samples) and were analyzed using one-way analysis of variance followed by *post hoc* Tukey’s multiple comparison test. *P < 0.05, **P < 0.01, ***P < 0.001. Arg-1: Arginase 1; C15: chemerin 15; Co: co-culture system; CON: control; IL: interleukin; iNOS: inducible nitric-oxide synthase; ns: not significant; OGD: oxygen-glucose deprivation; PBS: phosphate buffered saline; TNF-α: tumor necrosis factor-α.

To determine the optimal concentration of C15 *in vitro*, we first treated BV2 cells with different concentrations of C15, subjected the cells to the OGD, and measured the mRNA levels of IL-1β, TNF-α, and Arg-1. Real-time polymerase chain reaction analysis showed that IL-1β and TNF-α mRNA expression levels decreased at C15 concentrations of 500 ng/mL and 1000 ng/mL, with no statistically significant difference between the two conditions (**Additional Figure 2**). Significantly increased Arg-1 expression was observed in cells treated with 500 ng/mL C15. Therefore, 500 ng/mL of C15 was used for subsequent *in vitro* experiments. We next explored the impact of C15 on BV2 cell polarization and cytokine secretion. Flow cytometry analysis (**Figure 2A–D**) showed that exposure to OGD resulted in a high percentage of CD86^+^ cells (84.58% ± 2.53%), while CD86^+^ cells accounted for only 46.15% ± 2.78% of microglia in the OGD + C15 group. The OGD group had a smaller percentage of CD206^+^ cells compared with that of the OGD + C15 group (24.08% ± 2.52% *vs*. 68.30% ± 2.11%, respectively). Furthermore, enzyme-linked immunosorbent assay (ELISA) analysis demonstrated that pretreatment with C15 decreased secretion of the inflammatory factors IL-1β, IL-6, TNF-α, and iNOS but increased secretion of the anti-inflammatory mediators Arg-1 and IL-10 compared with the OGD or OGD + PBS groups (**Figure 2E–J**). These results suggested that C15 administration skewed microglia toward the M2 phenotype and suppressed the secretion of inflammatory factors, which inhibited inflammation following I/R injury.

To simulate the effect of microglial cells on neurons post-stroke, we established a co-culture system (Co) of BV2 and HT-22 cells and assessed HT-22 cell apoptosis using flow cytometry. **Additional Figure 3** presents a schematic illustration of the Transwell system, which allows for the diffusion of soluble molecules between BV2 and HT-22 cells. Flow cytometric analysis (**Figure 2K** and **L**) showed a remarkable increase in the rate of HT-22 cell apoptosis in the OGD (Co) group. When C15 was added to the upper chamber containing BV2 cells, the HT-22 cell apoptosis rate was 38.83% ± 0.61%, which was approximately 23% less than that seen in the OGD (Co) group. These results suggest that C15 modulates microglia-related inflammatory responses after ischemia, thus indirectly protecting neurons.

**Figure 3:**
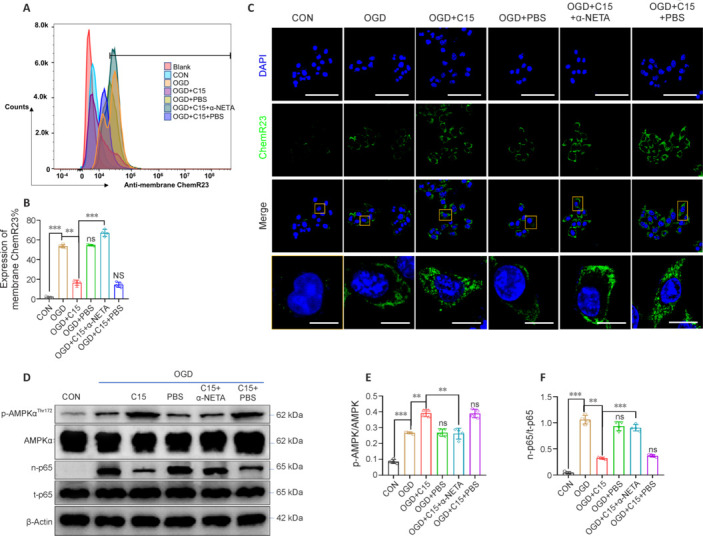
C15 induces ChemR23 internalization and regulates the AMPK/NF-κB signaling pathway. (A) Representative flow cytometric histograms showing that C15 treatment reduced ChemR23 expression on the membrane, whereas α-NETA reversed this effect. (B) The percentage of ChemR23 expressed on the cell membrane. (C) Digital fluorescence images showed increased intracellular accumulation of ChemR23 in the C15 group and a decreased tendency toward intracellular ChemR23 accumulation in the α-NETA group. The regions outlined in yellow are shown at higher magnification in the bottom row of images. Green, ChemR23; blue, DAPI. Scale bars: 50 μm, 10 μm (enlarged images). (D) Western blot showing increased p-AMPKThr172 expression and decreased nuclear NF-κB p65 expression in the C15 group; treatment with α-NETA reversed these effects. (E, F) Densitometric quantification of p-AMPK/AMPK (E) and n/t-p65 (F) expression. Data are presented as the mean ± SD (*n* = 4 independent samples) and were analyzed using one-way analysis of variance with *post hoc* Tukey’s multiple comparison test. **P < 0.01, ***P < 0.001. AMPK: Adenosine 5ʹ-monophosphate-activated protein kinase; C15: chemerin 15; ChemR23: chemokine-like receptor 1; CON: control; NF-κB: nuclear factor kappa B; n-p65: nucleus-p65; ns: not significant; OGD: oxygen-glucose deprivation; PBS: phosphate buffered saline; t-p65: total-p65; α-NETA: 2-(alpha-Naphthoyl) ethyltrimethylammonium iodide.

### C15 induces ChemR23 internalization and regulates the AMPK/NF-κB signaling pathway

ChemR23, a seven-transmembrane G protein–coupled receptor, is expressed in fat cells, dendritic cells, neutrophils, and tissue-resident macrophages. Previous studies reported that ChemR23 internalization activates cell signaling (Bondue et al., 2011; Zhou et al., 2014; Imaizumi et al., 2022); however, it remains unknown whether C15 promotes ChemR23 internalization.

To test this, we treated BV2 cells with C15 and α-NETA (a competitive inhibitor of ChemR23) prior to subjecting the cells to OGD and quantified membrane ChemR23 expression by cytometry and intercellular ChemR23 expression by immunofluorescence staining. *In vitro* flow cytometry (**Figure 3A**, **B**, and **Additional Figure 4**) showed that 1.56% ± 0.05% of ChemR23 was expressed on the membrane surface in the CON group. OGD increased ChemR23 expression on the surface in BV2 cells compared to the CON group, while treatment with C15 decreased cell-surface ChemR23 expression (54.18% ± 1.63% with OGD alone *vs*. 18.05% ± 0.88% with OGD + C15). Pretreating BV2 cells with α-NETA increased the percentage of ChemR23 expressed on the membrane surface to 68.68% ± 0.88%. Immunofluorescence staining (**Figure 3C**) showed that ChemR23 expression was markedly upregulated in the OGD group compared with the CON group, and ChemR23 was mainly located on the cell membrane rather than in the cytoplasm, after OGD. C15 treatment increased the intracellular accumulation of ChemR23 compared with the OGD group. As expected, α-NETA counteracted the effect of C15 on ChemR23 internalization.

**Figure 4:**
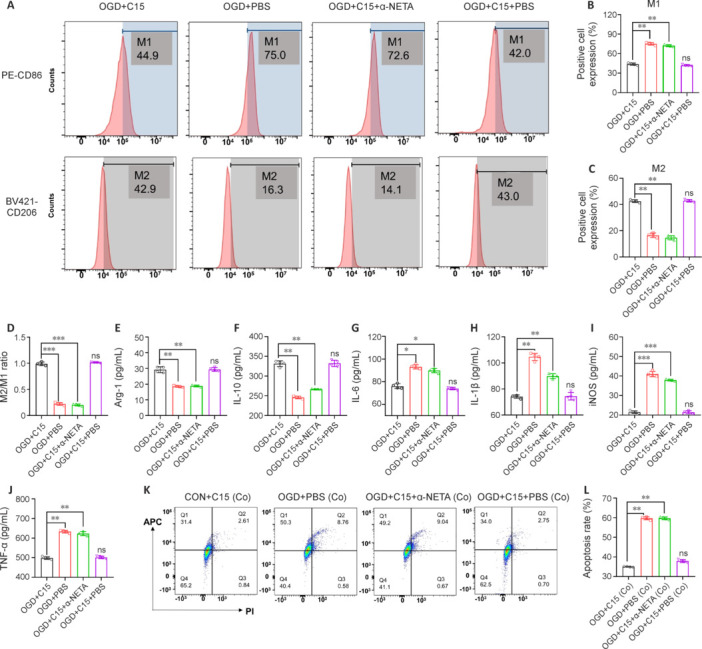
C15 exerts anti-inflammatory and neuroprotective effects through ChemR23. (A) Flow cytometry showed a higher level of M2 microglia and lower level of M1 microglia in the C15 group, and an opposite effect in the α-NETA group. (B–D) Quantitative analysis of the number of CD86^+^ cells (B) and CD206^+^ cells (C) and the ratio of M2 to M1 microglia (D). (E–J) ELISA analysis indicated that α-NETA administration counteracted the effects of C15, resulting in increased expression of pro-inflammatory factors (IL-1β, IL-6, iNOS, and TNF-α) and decreased expression of anti-inflammatory factors (Arg-1 and IL-10). (K) Fluorescent dot plot of HT-22 cell apoptosis, as assessed by flow cytometry. The apoptosis rate was markedly increased in the OGD + C15 + α-NETA (Co) group compared with the OGD + C15 (Co) group. (L) Quantification of HT-22 cell apoptosis. Data are presented as the mean ± SD (*n* = 4 independent samples) and were analyzed using one-way analysis of variance with *post hoc* Tukey’s multiple comparison test. *P < 0.05, **P < 0.01, ***P < 0.001. Arg-1: Arginase 1; C15: chemerin 15; Co: co-culture system; ELISA: enzyme-linked immunosorbent assay; IL: interleukin; iNOS: inducible nitric-oxide synthase; ns: not significant; OGD: oxygen-glucose deprivation; PBS: phosphate buffered saline; TNF-α: tumor necrosis factor-α; α-NETA: 2-(alpha-naphthoyl) ethyltrimethylammonium iodide.

We next investigated downstream signaling events after ChemR23 internalization. ChemR23 can continue signaling after internalization, together with its agonists, and trigger specific downstream effects (Wang et al., 2018a). Chemerin suppresses neuroinflammation by upregulating the ChemR23/AMPK signaling pathway (Zhang et al., 2018). Phosphorylated (active) AMPK helps inhibit NF-κB nuclear translocation (Salminen et al., 2011), thereby reducing the expression of inflammatory cytokines and protecting the brain from I/R injury. To gain mechanistic insights into the anti-inflammatory effects of C15 on BV2 cells, we analyzed the protein expression levels of components of the ChemR23/AMPK/NF-κB signaling pathway. Western blot analysis (**Figure 3D–F**) revealed that OGD induced the upregulation of p-AMPKThr172 and nuclear NF-κB p65 (n-p65), while total AMPK and NF-κB p65 (t-p65) expression levels remained relatively unchanged among the groups. In contrast, the increase in p-AMPKThr172 was more obvious following C15 administration, and the n-p65 level was reduced, suggesting that C15 promotes AMPK activation and reduces NF-κB p65 translocation into the nucleus through ChemR23. Treatment with α-NETA weakened this effect. In summary, ischemia-hypoxia stimulation induced ChemR23 expression in BV2 cells, and treatment with C15 triggered ChemR23 internalization, thereby regulating the AMPK/NF-κB signaling pathway.

### C15 suppresses microglia-mediated inflammation and promotes HT-22 cell survival via ChemR23 *in vitro*

C15 may exert its anti-inflammatory and neuroprotective effects via ChemR23. To assess this, we detected microglial phenotypes, cytokine expression levels, and apoptosis of neurons co-cultured with microglia. Flow cytometry (**Figure 4A–D**) analysis showed that there was a higher percentage of CD86^+^ cells (72.33% ± 1.50% with α-NETA *vs*. 44.13% ± 1.70% without α-NETA) and a lower percentage of CD206^+^ cells (14.65% ± 1.58% with α-NETA *vs*. 42.55% ± 1.09% without α-NETA) in the OGD + C15 + α-NETA group than the OGD + C15 group. Similarly, expression levels of the inflammatory factors IL-1β, IL-6, TNF-α, and iNOS increased, and those of the anti-inflammatory mediators Arg-1 and IL-10 decreased, in cells treated with α-NETA compared with untreated cells (**Figure 4E–J**). In addition, flow cytometry analysis indicated that the apoptotic rate of HT-22 cells was 35.03% ± 0.20% in the OGD + C15 (Co) group; however, when BV2 cells were treated with α-NETA, the apoptosis rate increased by approximately 24% compared with the OGD + C15 (Co) group (**Figure 4K** and **L**). These results confirm that C15 inhibits inflammation in BV2 cells and has a protective effect on neurons via ChemR23.

### C15 reduces infarct volume and neuronal apoptosis and regulates microglial phenotype via ChemR23 after ischemia-reperfusion injury

**Figure 5:**
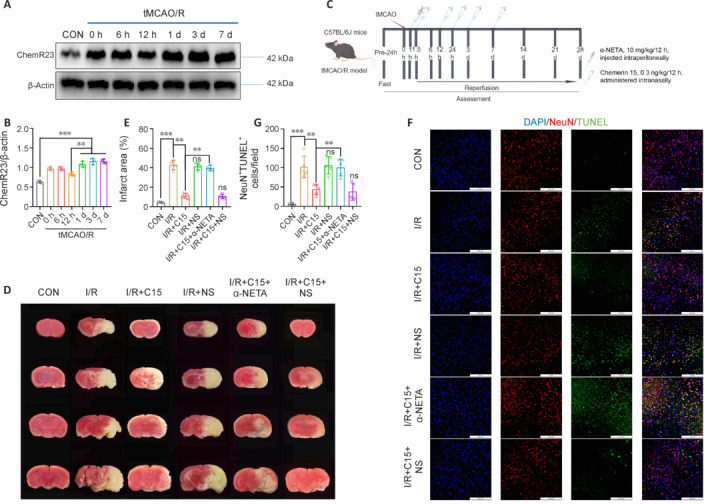
C15 decreases infarct volume and neuronal apoptosis via ChemR23 *in vivo.* (A) Representative western blot showing that ChemR23 expression was markedly upregulated 24 hours following tMCAO/R and plateaued at 3 and 7 days. (B) Densitometric quantification of ChemR23 expression (*n* = 3 independent samples). (C) Experimental timeline. (D) TTC staining showed that C15 administration reduced the infarct volume, while α-NETA reversed this effect. (E) Quantification of infarct volume based on TTC staining (*n* = 4, independent samples). (F) Representative images of nuclei (DAPI, blue), neurons (NeuN, Alexa Fluor® 647, red), and apoptotic cells (TUNEL, FITC, green). A large number of NeuN^+^TUNEL^+^ cells were observed in the I/R group that was decreased by C15 treatment and increased by α-NETA treatment through inhibition of ChemR23. Scale bars: 200 μm. (G) Semi-quantitative fluorescence analysis of NeuN^+^TUNEL^+^ cells (*n* = 5 independent samples). Data are presented as the mean ± SD and were analyzed using one-way analysis of variance with *post hoc* Tukey’s multiple comparison test. **P < 0.01, ***P < 0.001. C15: Chemerin 15; CON: control; DAPI: 4,6-diamidino-2-phenyl-indole; FITC: fluorescein isothiocyanate; IF: immunofluorescence; I/R: ischemia-reperfusion; NeuN: neuronal nuclei; ns: not significant; NS: normal saline; tMCAO/R: transient middle cerebral artery occlusion-reperfusion; TTC: 2,3,5-triphenyltetrazolium chloride staining; TUNEL: terminal deoxynucleotidyl transferase-mediated dUTP nick-end; α-NETA: 2-(alpha-naphthoyl) ethyltrimethylammonium iodide.

Considering the *in vitro* results described above, we investigated the anti-inflammatory and neuroprotective effects of C15 on mice subjected to I/R injury. To do this, we first assessed ChemR23 expression by western blot at 0, 6, and 12 hours and 1, 3, and 7 days following tMCAO/R. ChemR23 expression increased until 1 day after tMCAO/R, after which it plateaued (**Figure 5A** and **B**). Therefore, we investigated the protective effects of C15 on I/R injury and its interaction with ChemR23 at 24 hours post-injury.

Before subjecting the mice to tMCAO/R, they were treated with C15 intranasally, as shown in **Figure 5C**. We assessed the impact of C15 on infarct volume via 2,3,5-triphenyltetrazolium chloride staining. As shown in **Figure 5D** and **E**, the percentage of infarct volume out of the total brain volume was 42.76% ± 4.78% in the I/R group, confirming successful establishment of the tMCAO/R model. C15 treatment markedly decreased this percentage to 11.45% ± 2.53%, while α-NETA increased the percentage of infarct volume to 39.74% ± 2.80%.

We further explored the effect of C15 on neuronal apoptosis via *in vivo* double immunofluorescence staining. As shown in **Figure 5F** and **G**, the number of apoptotic neurons (NeuN^+^TUNEL^+^ cells) in the I/R group was significantly higher than that seen in the CON group, while the number of apoptotic neurons in the I/R + C15 group was decreased compared with the I/R group. The protective effect of C15 was weakened by treatment with α-NETA. No significant difference was observed in the number of apoptotic neurons between the I/R and I/R + C15 + α-NETA groups.

**Figure 6:**
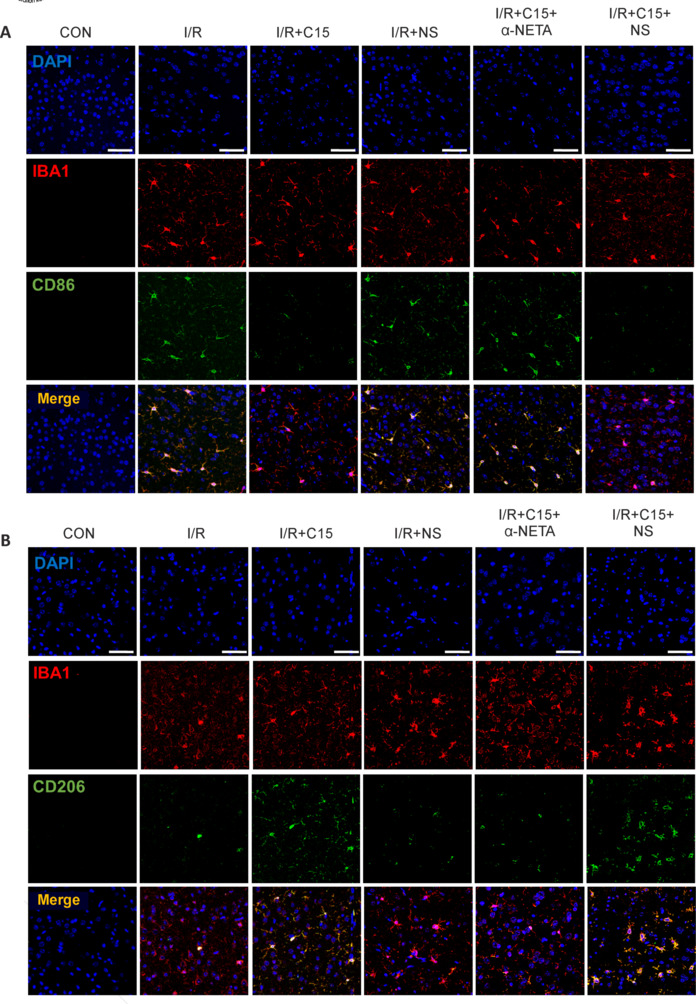
C15 regulates microglial phenotype through ChemR23 *in vivo*. (A) Fluorescence images of double staining for an activated microglia marker (IBA1, Alexa Fluor® 647, red) and an M1 microglia marker (CD86, Alexa Fluor® 488, green) (*n* = 5, independent samples). Fewer IBA1^+^CD86^+^ cells were observed in the peri-infarct area of the I/R + C15 group than in the I/R + C15 + α-NETA group. Scale bar: 50 μm. (B) Representative fluorescent images of double staining for an activated microglia marker (IBA1, Alexa Fluor® 647, red) and an M2 microglia marker (CD206, FITC, green) (*n* = 5, independent samples). The number of IBA1^+^CD206^+^ cells increased with C15 administration, and only a small number of IBA1^+^CD206^+^ cells were observed in the I/R + C15 + α-NETA group. Scale bar: 50 μm. C15: Chemerin 15; CON: control; DAPI: 4,6-diamidino-2-phenyl-indole; FITC: fluorescein isothiocyanate; I/R: ischemia-reperfusion; IBA1: ionized calcium-binding adapter molecule 1; tMCAO/R: transient middle cerebral artery occlusion/reperfusion; α-NETA: 2-(alpha-naphthoyl) ethyltrimethylammonium iodide.

Subsequently, we conducted immunofluorescence staining to assess microglial phenotypes. As shown in **Figure 6A** and **B**, normal brain tissue was almost devoid of IBA1^+^ cells (activated microglia). The number of IBA1^+^ cells in the peri-infarct area was increased in the I/R group, demonstrating activation of resting microglia following I/R injury. A large number of IBA1^+^CD206^+^ cells (M2 microglia) and a few IBA1^+^CD86^+^ cells (M1 microglia) were observed in the peri-infarct area in the I/R + C15 group. Intraperitoneal injection of α-NETA led to a remarkable decrease in the number of IBA1^+^CD206^+^ cells and an increase in the number of IBA1^+^CD86^+^ cells. These results imply that C15 reduces infarct volume and neuronal apoptosis and modulates microglial polarization through ChemR23 in mice subjected to I/R injury.

### C15 regulates the expression of inflammatory cytokines and improves long-term outcomes in mice subjected to ischemia-reperfusion injury

**Figure 7:**
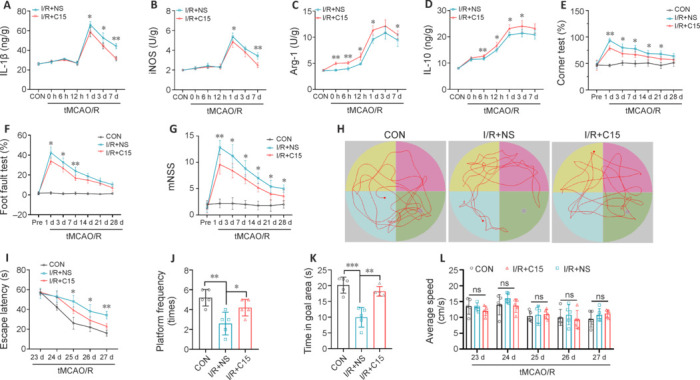
C15 regulates inflammatory cytokines and improves long-term outcome *in vivo.* (A–D) Statistical analysis of the expression of the pro-inflammatory cytokines IL-1β (A) and iNOS (B) and the anti-inflammatory cytokines Arg-1 (C) and IL-10 (D) at 0, 6, and 12 hours and 1, 3, and 7 days in the I/R + NS and I/R + C15 groups, as determined by ELISA. C15 increased the secretion of anti-inflammatory cytokines (Arg-1 and IL-10) in the early stage after injury (6 hours) and reduced the expression of pro-inflammatory cytokines (IL-1β and iNOS) at 24 hours. (E–G) Neurological function was evaluated using the corner test (E), foot fault test (F), and mNSS scoring system (G). The I/R + C15 group exhibited fewer faults and lower scores, suggesting that C15 improved neurological deficits. (H–L) Cognitive function was evaluated by Morris water maze test. (H) Representative images of swimming paths 28 days after tMCAO/R. (I) Quantification of escape latency in the training test. The average escape latency was shorter in the I/R + C15 group than in the I/R + NS group. (J–L) Quantitative analysis of the probe test results, including platform frequency (J), time spent in the target area (K), and average swimming speed (L) (*n* = 5 independent samples). Both the platform frequency and the time spent in target area were increased after C15 treatment, while the average swimming speed did not differ among the groups over 5 days. Data are presented as the mean ± SD (*n* = 5 independent samples) and were analyzed using two tailed *t*-test between two groups and repeated measurement analysis of variance and one-way analysis of variance with *post hoc* Tukey’s multiple comparison test among multiple two groups. *P < 0.05, **P < 0.01, ***P < 0.001. Arg-1: Arginase 1; C15: chemerin 15; CON: control; ELISA: enzyme-linked immunosorbent assay; I/R: ischemia-reperfusion; IL: interleukin; iNOS: inducible nitric-oxide synthase; mNSS: modified neurological severity score; ns: not significant; NS: normal saline; tMCAO/R: transient middle cerebral artery occlusion/reperfusion.

Next, we used ELISA kits to detect IL-1β, iNOS, IL-10, and Arg-1 secretion at different time points following tMCAO/R. The results showed that expression of the inflammatory factors IL-1β and iNOS increased following I/R injury, peaking at 24 hours, and decreased over time. C15 administration significantly decreased expression of the inflammatory mediators IL-1β and iNOS and promoted their swift return to baseline levels. Expression levels of the anti-inflammatory mediators Arg-1 and IL-10 increased notably with C15 administration, and this effect lasted until 7 days post-injury. Surprisingly, Arg-1 expression increased earlier in the group treated with C15 than in the I/R group (**Figure 7A–D**). These results suggest that C15 reduced the levels of inflammatory factors and promoted resolution of inflammation in the early stages following I/R injury, and that these effects might last a long time.

To verify the long-term effects of C15 on I/R mice, we used the foot fault test, corner turn test, and mNSS scoring system to evaluate motor function, sensory function, and balance, respectively. Compared with the control group, the mice in the I/R group exhibited neurological impairment. Mice treated with C15 demonstrated a lower percentage of foot faults, less side preference in the corner turn test, and lower mNSS scores (**Figure 7E–G**).

To assess the spatial learning ability and memory of I/R mice, we conducted the Morris water maze test (**Figure 7H–L**). In the training phase of the test, C15 treatment decreased the escape latency, especially on day 28, compared with the I/R + normal saline group. In the probe test, the I/R mice rarely crossed the target area, while the I/R + C15 mice crossed the platform frequently. Quantitative analysis showed that the mice in the I/R + C15 group exhibited a higher frequency and longer time in the target area than the I/R + normal saline group, suggesting that C15 improved spatial learning and memory function. There was no statistically significant difference in average swimming speed among the three groups. These results indicate that C15 treatment exerts long-term protective effects following I/R injury.

## Discussion

In this study, we investigated the effect of C15 on neuroinflammation and elucidated the potential underlying mechanisms. The main findings were as follows: (1) C15 skewed microglia polarization toward the M2 phenotype, reduced the expression of pro-inflammatory cytokines, and protected HT-22 cells from BV2-mediated cytotoxicity following OGD; (2) C15 bound to ChemR23 on the surface of microglia and induced its internalization, thereby regulating the AMPK/NF-κB signaling pathway; (3) C15 ameliorated ischemic brain injury and neurological deficits and modulated microglia polarization in mice subjected to I/R injury.

Inflammation is involved in the acute and chronic phases of injury following ischemic stroke and determines the degree of damage and patient prognosis (Endres et al., 2022; Qi et al., 2024; Li et al., 2025; Zheng et al., 2025). Persistent inflammation leads to long-term sequelae such as post-stroke depression, dementia, seizures, and fatigue (Shi et al., 2019; Endres et al., 2022; Huo et al., 2024). Microglia play biphasic roles in this process in response to different microenvironment stimuli. We observed dynamic changes in microglial polarization over time following OGD: the pro-inflammatory M1 phenotype and cytokines dominated at an early stage, followed by a gradual increase in the anti-inflammatory M2 phenotype and mediators. The ischemic mice model exhibited neuroinflammation, neuronal apoptosis, and neurological deficits in response to I/R injury. Terminating acute inflammatory responses to avoid chronic inflammation and sequelae is an ideal therapeutic goal. Pro-resolving mediators (e.g., chemerin, Annexin A1, Resolvin E1) are the best candidates for this type of treatment, as they actively inhibit inflammation. Pro-resolving mediators eliminate inciting stimuli, inhibit pro-inflammatory signals, promote pro-inflammatory mediator catabolism, and restore tissue function (Feehan and Gilroy, 2019).

Chemerin and its derivatives are important pro-resolving mediators (Mariani and Roncucci, 2015). However, in addition to inhibiting the inflammatory response, they have moderate chemotactic activity that may aggravate the inflammatory response (Sato et al., 2019; Chen et al., 2022). Chemerin 15 is the only known chemerin polypeptide with potent anti-inflammatory effects that does not exhibit chemotactic activity at picomolar concentrations, while other fragments are active at nanomolar concentrations. C15 inhibits neutrophil recruitment and infiltration, reduces the secretion of pro-inflammatory cytokines, skews macrophages to a protective phenotype, and promotes phagocytosis of bacterial particles and apoptotic cells (Cash et al., 2010, 2013; Chang et al., 2015). Our findings suggest that C15 also decreases inflammation in the early stages after ischemic brain injury by shifting microglial polarization toward the M2 phenotype *in vivo* and *in vitro* and promoting the production of anti-inflammatory mediators. Furthermore, we found that C15 treatment indirectly protected neurons, reduced infarct volume, and improved long-term outcomes. Our results highlight the potential advantage of using C15 to inhibit the inflammatory response at an early stage, thus evading further neuronal damage and neurological impairment.

Three G protein–coupled receptors have been identified that bind to chemerin: the natural receptor ChemR23, chemokine CC motif receptor-like 2, and G protein–coupled receptor 1 (Su et al., 2021). ChemR23 has a wide variety of biological activities that have been described *in vitro* and *in vivo* (Xie and Liu, 2022). Chemerin binds to G protein–coupled receptor 1 with lower affinity and is involved in glucose homeostasis regulation (Kennedy and Davenport, 2018). Chemokine CC motif receptor-like 2, an “atypical” chemoattractant receptor devoid of ligand-scavenging properties, conjugates with ChemR23-expressing cells and regulates chemerin concentrations (Del Prete et al., 2013). Unlike chemerin, C15 engages ChemR23 to have anti-inflammatory effects without exerting chemotactic activity. ChemR23 knockout has been shown to abrogate the anti-inflammatory effect of C15 but only reduce the anti-inflammatory activity of chemerin (Cash et al., 2008). Our results are consist with previous study showing that ChemR23 was rapidly upregulated upon inflammatory cell activation (Cash et al., 2013). Our internalization experiments showed that C15 promoted uptake of ChemR23 receptors, and this effect was eliminated by treatment with α-NETA (a competitive inhibitor of ChemR23). These results suggest that C15 binds to ChemR23 and promotes its internalization.

We further investigated the molecular mechanism by which C15 resolves inflammation following ischemic injury. Our study showed that OGD increased BV2 cell expression of p-AMPKThr172 and n-p65. This may be because AMPK is activated in response to various cellular stresses, including hypoxia, inflammation, nutrient deprivation, and oxidative stress (Zhu et al., 2016; Chen et al., 2018). The α-subunit of AMPK contains Thr172, the phosphorylation of which is required for AMPK activation (Herzig and Shaw, 2018). AMPK signaling reduces inflammation by inhibiting the activation of downstream NF-κB signaling (Salminen et al., 2011). Activation of canonical NF-κB signaling allows for the release of NF-κB dimers (p65 and p50) into the nucleus, where they rapidly, yet transiently, activate the transcription of pro-inflammatory genes (Yu et al., 2020). We next treated BV2 cells with C15 and observed a more significant increase in p-AMPKThr172 levels and nuclear translocation of NF-κB p65. This may be explained by chemerin-mediated activation of the AMPK pathway affecting downstream signaling molecules, as demonstrated in previous studies (Wang et al., 2018b; Jian et al., 2019; Xu et al., 2021). Furthermore, α-NETA inhibited ChemR23 internalization and partially reversed the changes caused by C15. These findings indicate that ChemR23 internalization may affect the AMPK/NF-κB signaling pathway, which participates in the C15-mediated resolution of microglial inflammation.

This study had some limitations. First, we focused on the anti-inflammatory and neuroprotective effects of C15 on microglia; however, ChemR23 is also expressed by other neural cells (e.g., astrocytes) and infiltrating peripheral immune cells that influence stroke outcomes. Therefore, the effect of C15 on these cells and their interactions remain to be fully investigated. In addition, we only provide primary evidence regarding the therapeutic role of C15; therefore, future studies should develop C15 delivery systems to achieve stable blood concentrations and determine optimal therapeutic doses in animals prior to clinical trials.

In conclusion, the findings from this study suggested that C15 promotes resolution of inflammation by skewing microglial polarization toward an anti-inflammatory and protective phenotype, reducing the expression pro-inflammatory cytokines, protecting neurons against microglia-induced inflammation, and alleviating neurological deficits. These effects are associated with ChemR23 internalization and the AMPK/NF-κB signaling pathway and demonstrate the potential role of C15 in protecting against cerebral I/R injury.

## Additional files:

***Additional Figure 1:*** *BV2 cell viability after OGD.*

Additional Figure 1 BV2 cell viability after OGD.
CCK-8 assay showing that the percentage of viable BV2 cells decreased to approximately 50% at 3 hours following OGD. Data are expressed as mean ± SD (*n* = 4 independent samples). CCK-8: Cell Counting Kit-8; CON: control; OGD: oxygen glucose deprivation.

***Additional Figure 2:*** *Effect of different C15 concentrations on cytokine expression in vitro.*

Additional Figure 2 Effect of different C15 concentrations on cytokine expression *in vitro*.
Real-time polymerase chain reaction analysis of IL-1β, TNF-α, and Arg-1 expression levels in BV2 cells treated with different concentrations of C15. At a concentration of 500 ng/mL, the mRNA expression levels of IL-1β and TNF-α were clearly decreased, while the expression level of Arg-1 was markedly increased. Data are presented as the mean ± SD (*n* = 3 independent samples) and were analyzed using one-way analysis of variance with *post hoc* Tukey's multiple comparison test. *P < 0.05, **P < 0.01. Arg-1: Arginase 1; C15: chemerin 15; IL: interleukin; TNF-α: tumor necrosis factor-α.

***Additional Figure 3:*** *Schematic illustration of the Transwell system.*

Additional Figure 3 Schematic illustration of the Transwell system.
A BV2 and HT-22 cell co-culture system following OGD was established to simulate the effects of microglia on neurons after ischemic stroke. Created with FigDraw.com. OGD: Oxygen glucose deprivation.

***Additional Figure 4:*** *Cell membrane ChemR23 expression in the different groups.*

Additional Figure 4 Cell membrane ChemR23 expression in the different groups.
(A) Flow cytometric analysis of ChemR23 expression on the surface of BV2 cells (blank as an example). (B-G) Histogram of cell membrane ChemR23 expression in the different groups. C15: Chemerin 15; CON: control; OGD: oxygen glucose deprivation; PBS: phosphate buffered saline; α-NETA: 2-(alpha-Naphthoyl) ethyltrimethylammonium iodide.

## Data Availability

*All data relevant to the study are included in the paper or uploaded as Additional files*.
