# Supplementary figures and images for: Chemerin 15 peptide reduces neuroinflammation via the ChemR23 receptor after ischemia–reperfusion injury
Source: Neural Regen Res. 2024 Sep 6;21(7):2986–96. doi: 10.4103/NRR.NRR-D-24-00137 (PMC13378918; doi:10.4103/NRR.NRR-D-24-00137)

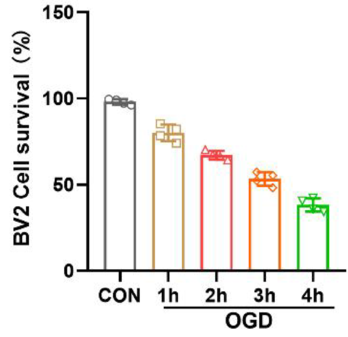

Supplement: Supplementary file 1 [file NRR-21-2986_Suppl1.tif]

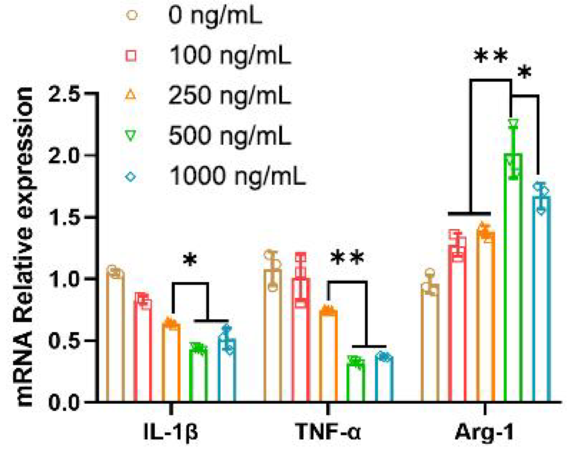

Supplement: Supplementary file 2 [file NRR-21-2986_Suppl2.tif]

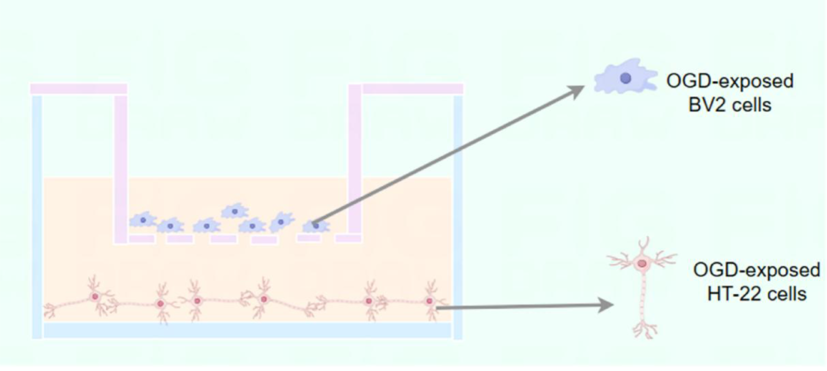

Supplement: Supplementary file 3 [file NRR-21-2986_Suppl3.tif]

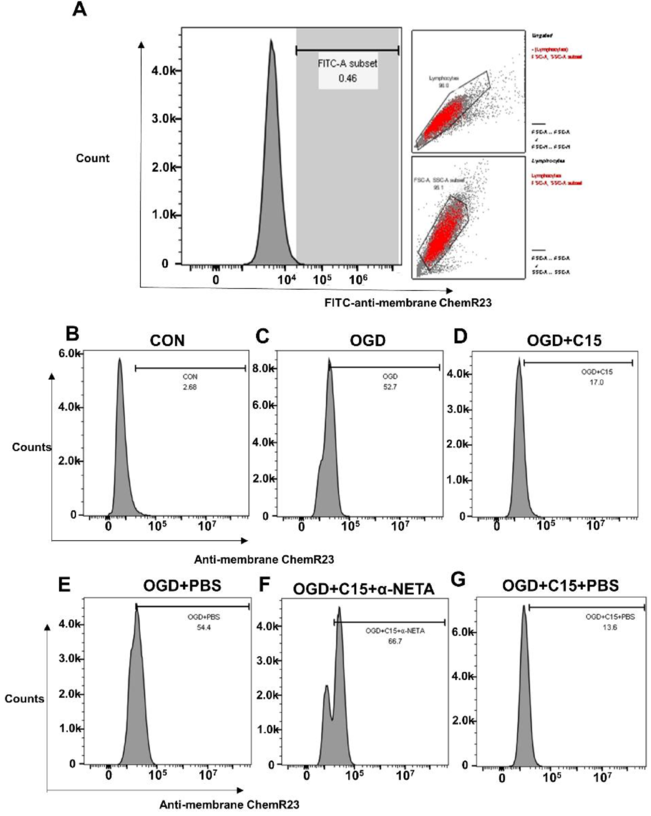

Supplement: Supplementary file 4 [file NRR-21-2986_Suppl4.tif]
